# Using human disease mutations to understand *de novo* DNA methyltransferase function

**DOI:** 10.1042/BST20231017

**Published:** 2024-10-24

**Authors:** Willow Rolls, Marcus D. Wilson, Duncan Sproul

**Affiliations:** 1 MRC Human Genetics Unit, Institute of Genetics and Cancer, University of Edinburgh, Edinburgh, U.K.; 2 Wellcome Centre for Cell Biology, University of Edinburgh, Edinburgh, U.K.; 3 CRUK Edinburgh Centre, Institute of Genetics and Cancer, University of Edinburgh, Edinburgh, U.K.

**Keywords:** cancer, epigenetics, genetic disease, methylation

## Abstract

DNA methylation is a repressive epigenetic mark that is pervasive in mammalian genomes. It is deposited by DNA methyltransferase enzymes (DNMTs) that are canonically classified as having *de novo* (DNMT3A and DNMT3B) or maintenance (DNMT1) function. Mutations in DNMT3A and DNMT3B cause rare Mendelian diseases in humans and are cancer drivers. Mammalian DNMT3 methyltransferase activity is regulated by the non-catalytic region of the proteins which contain multiple chromatin reading domains responsible for DNMT3A and DNMT3B recruitment to the genome. Characterising disease-causing missense mutations has been central in dissecting the function and regulation of DNMT3A and DNMT3B. These observations have also motivated biochemical studies that provide the molecular details as to how human DNMT3A and DNMT3B mutations drive disorders. Here, we review progress in this area highlighting recent work that has begun dissecting the function of the disordered N-terminal regions of DNMT3A and DNMT3B. These studies have elucidated that the N-terminal regions of both proteins mediate novel chromatin recruitment pathways that are central in our understanding of human disease mechanisms. We also discuss how disease mutations affect DNMT3A and DNMT3B oligomerisation, a process that is poorly understood in the context of whole proteins in cells. This dissection of *de novo* DNMT function using disease-causing mutations provides a paradigm of how genetics and biochemistry can synergise to drive our understanding of the mechanisms through which chromatin misregulation causes human disease.

In mammals, DNA methylation predominantly occurs on the cytosines of CpG dinucleotides [1] and is thought to facilitate the repression of some gene promoters and retrotransposons [2,3]. DNA methylation is catalysed by DNA methyltransferase enzymes (DNMTs) [4]. Its genomic pattern is established during early development but varies between tissues and cell types [5]. This establishment is largely accomplished by the *de novo* DNMTs DNMT3A and DNMT3B [4]. Thereafter DNA methylation patterns are thought to be maintained primarily by DNMT1 [6] with assistance of its recruitment factor UHRF1 [7–9]. Consistent with this idea, DNMT1 preferentially methylates hemi-methylated DNA *in vitro* [10] which is generated by DNA replication. In contrast, DNMT3A and DNMT3B lack this preference [11] but also contribute to DNA methylation maintenance [12–14]. Removal of DNA methylation occurs through both passive dilution following replication or TET-enzyme mediated active demethylation [15,16].

Aberrant DNA methylation patterns develop during aging and in cancers [17–20]. *DNMT3A* and *DNMT3B* mutations cause human Mendelian disorders and somatic driver mutations are reported in cancer (Table 1). Here, we review how the study of disease-causing *DNMT3A* and *DNMT3B* mutations has advanced our understanding of the molecular mechanisms regulating *de novo* DNMT activity.

**Table 1.** Summary of human diseases associated with DNMT3 mutations discussed in this article.

Disease	Gene	Type of mutation	Phenotype	Type of DNA methylation change
Tatton-Brown-Rahman syndrome (TBRS) [21] OMIM: 615879	DNMT3A	Germline heterozygous loss of function	Overgrowth and intellectual disability	Loss
Heyn-Sproul-Jackson syndrome (HESJAS) [22] OMIM: 618724	DNMT3A	Germline heterozygous gain of function	Microcephalic dwarfism and global developmental delay	Gain
Immunodeficiency centromeric instability and facial anomalies syndrome type 1 (ICF1): [23] OMIM: 242860	DNMT3B	Germline homozygous loss of function	Immunodeficiency, facial dysmorphism, chromosomal abnormalities	Loss
Acute myeloid leukaemia (AML) [24]	DNMT3A	Somatic heterozygous loss of function	Cancer	Loss
Paraganglioma [25]	DNMT3A	Somatic and germline heterozygous gain of function	Cancer	Gain
Prostate cancer [26]	DNMT3B	Somatic heterozygous unknown	Cancer	Gain

OMIM, Online Mendelian Inheritance in Man accession.

## Human *de novo* DNA methyltransferases

**Figure 1.**
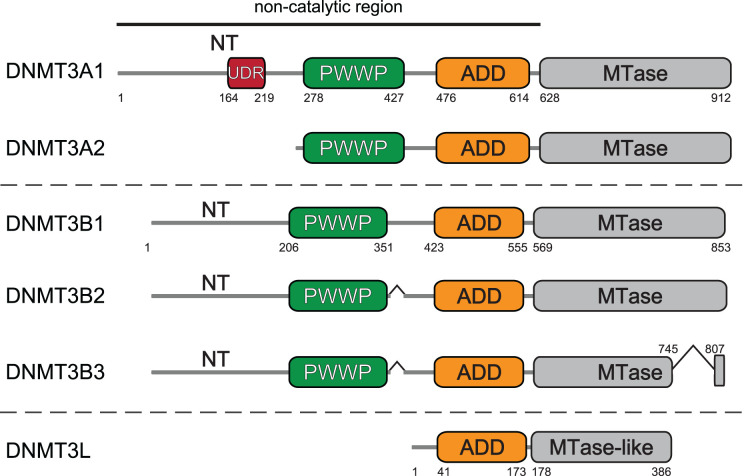
Human DNMT3 enzyme domain structure. Schematic showing annotated domain structure of canonical human DNMT3 protein isoforms. The two isoforms of DNMT3A are produced by alternative promoter usage. The three isoforms for DNMT3B are produced by alternative splicing. Abbreviations used: NT, N-terminal region; UDR, ubiquitin-dependent recruitment region; PWWP, Pro-Trp-Trp-Pro domain; ADD, ATRX-DNMT3-DNMT3L domain; MTase, methyltransferase domain. Numbers below schematics indicate position in residues.

DNMT3A and DNMT3B possess a C-terminal catalytic methyltransferase domain and two chromatin reading domains; an ADD (ATRX-Dnmt3-Dnmt3L) domain and a PWWP (Pro-Trp-Trp-Pro) domain (Figure 1). They also have an unstructured N-terminal region (Figure 1). Despite their similar domain structure, they are not functionally redundant. In mice, *DNMT3A* knockout is lethal postnatally whereas *DNMT3B* knockouts are inviable past early gestation [4].

**Figure 2.**
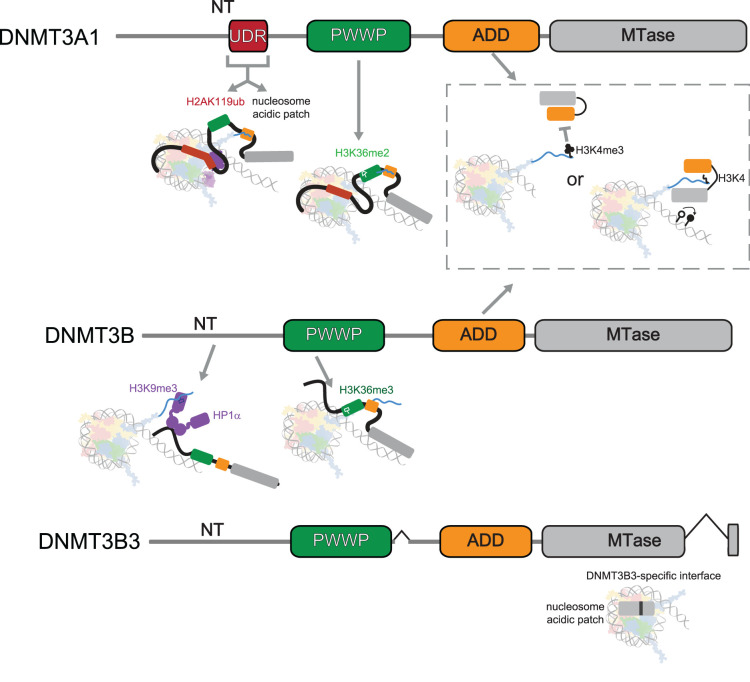
Human DNMT3 enzyme chromatin reading activities. Schematic summarising interactions between human DNMT3 enzymes and chromatin features that are proposed to regulate or recruit the enzymes. The UDR of DNMT3A binds H2AK119ub and the nucleosome acidic patch on the nucleosome surface between H2A and H2B [27–29]. The PWWP domain of DNMT3A binds H3K36me2 whereas that of DNMT3B binds H3K36me3 [30,31]. H3K4me3 prevents binding of the ADD domain to chromatin, so instead the ADD binds to the methyltransferase domain and auto-inhibits the enzyme [32,33]. In the absence of H3K4 methylation, the ADD domain can bind H3K4, which relieves the auto-inhibition and allows the methyltransferase domain to methylate DNA. The N-terminal region of DNMT3B can bind to HP1α, which binds to H3K9me3 [34]. Splicing of the DNMT3B3 isoform forms a new interface that binds to the acidic patch on the nucleosome [35]. Abbreviations used: NT, N-terminal region; UDR, ubiquitin-dependent recruitment region; PWWP, Pro-Trp-Trp-Pro domain; ADD, ATRX-DNMT3-DNMT3L domain; MTase, methyltransferase domain.

The non-catalytic region of DNMT3A and DNMT3B regulate their activity and recruitment to the genome. This primarily occurs through the recognition of specific chromatin features and histone modifications (Figure 2) which are a strong determinant of DNA methylation patterns in the genome, as recently extensively reviewed [36–38].

*De novo* methyltransferase function is also regulated through expression of alternative isoforms. There are two isoforms of DNMT3A: DNMT3A1 is the full-length, somatic isoform and a shorter isoform lacking the N-terminal region, DNMT3A2, is primarily expressed in stem and germ cells [39–42] (Figure 2). DNMT3B has 3 canonical isoforms, DNMT3B1, DNMT3B2 and DNMT3B3 [43] (Figure 2). DNMT3B1 and DNMT3B2 are reported to be expressed in stem and somatic cells respectively and differ by an internal exon between the PWWP and ADD domain [44]. DNMT3B3 is catalytically inactive as it lacks two exons of the methyltransferase domain [44–46]. Many other DNMT3B isoforms have been reported to be expressed in cancers [47,48] but are poorly characterised, making their functional impact and expression levels unclear. DNMT3B3 functions as an adaptor protein that stimulates DNMT3A and DNMT3B activity [35,49–51]. DNMT3L plays a similar role during early development and the germline and is required to establish DNA methylation patterns [52–56].

**Figure 3.**
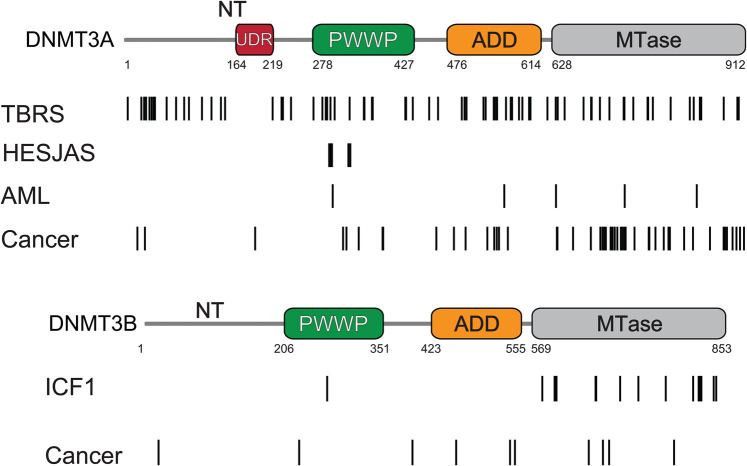
Distribution of disease mutations in human DNMT3 enzymes. (**A**) *DNMT3A* missense mutations from selected diseases. Schematic illustrating the distribution of disease mutations across the DNMT3A protein. Lines indicate individual disease mutations. TBRS, Tatton-Brown-Rahman syndrome, mutations taken from UniProt; HESJAS, Heyn-Sproul-Jackson syndrome, mutations taken from ClinVar filtered on pathogenic/likely-pathogenic classification [57]. AML, acute myeloid leukaemia, mutations taken from ClinVar filtered on pathogenic/likely-pathogenic classification [57]. Cancer mutations in DNMT3A taken from COSMIC with count ≥5 [58]. (**B**) *DNMT3B* missense mutations from selected diseases. Schematic illustrating the distribution of disease mutations across the DNMT3B protein. ICF1, immunodeficiency-centromeric instability-facial anomalies type 1, mutations taken from ClinVar pathogenic/likely-pathogenic classification [57]. Cancer mutations in *DNMT3B* taken from COSMIC with count ≥5 [58]. In both panels, lines indicate individual disease mutations. Numbers below schematics indicate position in residues. Abbreviations used on protein schematics: NT, N-terminal region; UDR, ubiquitin-dependent recruitment region; PWWP, Pro-Trp-Trp-Pro domain; ADD, ATRX-DNMT3-DNMT3L domain; MTase, methyltransferase domain.

Germline mutations in *DNMT3A* and *DNMT3B* cause different rare genetic diseases (Table 1). There are currently no reported disease-causing mutations in *DNMT3L*. *DNMT3A* disease-causing germline mutations are spread throughout the protein, while those in *DNMT3B* are almost exclusively found in the methyltransferase domain (Figure 3).

The overgrowth syndrome, Tatton-Brown-Rahman syndrome (TBRS), is caused by heterozygous loss-of-function mutations in *DNMT3A* [21]. TBRS patients display focal hypomethylation at developmental genes normally associated with regulation by Polycomb repressive complexes [59,60]. In contrast, heterozygous *DNMT3A* gain-of-function mutations cause Heyn-Sproul-Jackson syndrome (HESJAS) [22] which is characterised by microcephalic dwarfism and hypermethylation of Polycomb-marked DNA methylation valleys [22,61]. In the case of *DNMT3B*, homozygous loss-of-function mutations cause Immunodeficiency Centromeric instability and Facial anomalies syndrome type 1 (ICF1) [23] which displays chromosome instability and hypomethylation of constitutive heterochromatin [62,63].

Putative *DNMT3A* and *DNMT3B* somatic driver mutations are also widespread in cancer (Figure 3) but the majority of these are uncharacterised with the exception of *DNMT3A* mutations in acute myeloid leukeamia (AML). These loss-of-function mutations overlap with TBRS mutations and also show focal hypomethylation [60,64–66]. Germline and somatic *DNMT3A* gain-of-function mutations are also associated with paraganglioma [25]. *DNMT3B* mutations are more rarely reported in cancer (Figure 3) but are seen in advanced prostate cancers displaying a CpG island methylator phenotype [26]. While some disease-causing mutations are non-sense mutations [60,67], here we focus on missense mutations as they have been particularly fruitful in driving forward our understanding of DNMT3A and DNMT3B regulation.

## Domains and insights from biochemistry and disease

### Methyltransferase domain

The C-terminal methyltransferase domain of DNMT3A and DNMT3B catalyses methylation of cytosines [68]. It contains a catalytic loop that binds the cofactor *S*-adenosyl methionine (SAM) together with the cytosine substrate [69,70] and a central cysteine provides the nucleophile for catalysis [71]. The methyltransferase domain is the region of greatest amino acid homology between DNMT3A and DNMT3B, however they have different preferences for sequences flanking targeted CpGs [72–75]. This is mediated by target recognition loops that contact DNA differently [76]. Mutations to this loop alter their sequence specificity [72,76]. DNMT3A and DNMT3B can also methylate Cs occurring in a non-CpG context, with DNMT3A preferring CAC and DNMT3B preferring CAG [76–79]. Methylation of CA deposited by DNMT3A is abundant in the brain [80–83].

Missense mutations in *DNMT3A*’s methyltransferase domain are frequently observed in TBRS and AML (Figure 3) [21,24]. Many of these destabilise the protein or reduce its catalytic activity in cells [84]. 60% of AML mutations occur at Arg-882, with R882H observed most frequently [85]. The same residue is also mutated in TBRS [59]. Arg-882 interacts with the DNA backbone [70] and R882H changes the dynamics of the target recognition loop, altering the flanking sequence preference of DNMT3A *in vitro* [86,87]. This makes it similar to that of DNMT3B and emphasises the importance of this residue for flanking sequence preference and methyltransferase function. However, R882H has pleiotropic effects. It reduces DNMT3A catalytic activity [65,86,87] and its introduction into cells leads to hypomethylation suggesting it acts in a dominant negative fashion [64]. Several other AML-associated catalytic domain mutations including V716D, R792H and K841E are also reported to exhibit dominant negative effects [70]. These dominant negative effects chiefly occur due to methyltransferase domain-mediated oligomerisation of DNMT3A which will be discussed below.

Most ICF1 missense mutations occur in DNMT3B's methyltransferase domain (Figure 2) [67] and DNMT3B's consensus sequence also has a high frequency in the pericentromeric satellite 2 repeats hypomethylated in ICF1 [76]. Several ICF1 mutations are located within the DNMT3B methyltransferase structural core and are predicted to destabilise the protein [88]. Other ICF1 mutations frequently reduce DNMT3B's catalytic activity [71,89,90]. The ICF1 R823G mutation affects the residue paralagous to Arg-882 in DNMT3A. This mutation alters target recognition loop dynamics, and was recently reported to lower DNA binding affinity, alter flanking-sequence preference and reduce catalytic activity [91]. The R823A mutation also altered DNMT3B flanking-sequence specificity in an study of DNMT3B methyltransferase domain function [72]. However, another study reported R823G does not alter DNMT3B catalytic activity but instead leads to a DNA binding release defect [89] making the impact of this ICF1 mutation unclear.

### ADD domain

The ADD domains of DNMT3A and DNMT3B binds H3K4 [92–95]. When unliganded, the ADD inhibits access of the catalytic site to DNA [92] and binding to unmodified H3K4 alters DNMT3A conformation, releasing inhibition [94,96]. This allosteric activation is prevented by methylated H3K4 [32,33] which marks active promoters [97]. DNMT triple-knockout mouse embryonic stem cells (ESCs) expressing DNMT3A engineered to be insensitive to H3K4 methylation accumulate DNA methylation at H3K4me2/3 regions and differentiate abnormally [98]. Mice carrying DNMT3A D529A and D531A mutations that disrupt autoinhibition and H3K4me0 binding [92] have decreased CpG and non-CpG methylation in gametes [99,100] and have a dwarfism phenotype [99,100]. This demonstrates that ADD-mediated regulation of DNMT3A is developmentally important.

Although ADD mutations are reported in TBRS and occur near the H3 binding region [21], *DNMT3A* ADD disease mutations are less studied than those in other domains. In a systematic analysis of 253 *DNMT3A* TBRS or AML mutations, ADD mutations had milder effects on catalytic function and stability in cells than mutations in other domains [84]. Two residues mutated in AML, R556E and E907K, occur at the interface between the ADD-methyltransferase domain when DNMT3A is in its active confirmation [101]. These reduce activity, suggesting that the interaction between the ADD and methyltransferases domains remains important for DNMT3A function even after release of auto-inhibition by H3K4me0 [101].

A structural study reported that DNMT3B has weaker ADD-methyltransferase domain interaction than DNMT3A and the ADD adopts an extended conformation even in the absence of the H3 tail [102]. However, a study of DNMT3B's PWWP-ADD-methyltransferase domain observed that although DNMT3B's ADD domain has an alternate interaction with the methyltransferase domain compared with that of DNMT3A, it still possesses auto-inhibitory function [103]. In this study DNMT3B ADD-methyltransferase interaction was stabilised by the PWWP domain [103], potentially explaining the discrepancy between the two studies [102]. Furthermore, DNA methylation is anti-correlated with H3K4me3 in cells and when exogenous DNMT3B is introduced in *Saccharomyces cerevisiae* [104,105] strongly suggesting that DNMT3B's ADD possesses a similar autoinhibitory activity to the DNMT3A ADD *in vivo*.

There are currently no *DNMT3B* ADD mutations reported in ICF1 (Figure 2) [67]. However, two mutations of *DNMT3B*’s ADD have been observed in prostate cancer, E515D and R545C [26]. Tumours with these mutations have a CpG island methylator phenotype similar to those with TET2, IDH1 or BRAF mutations [26] suggesting they could affect DNMT3B regulation. However, at present they are functionally uncharacterised.

### PWWP domain

DNMT3A and DNMT3B are recruited to methylated H3K36 by an aromatic cage found in their PWWP domains [30,105–107]. DNMT3A's PWWP recognises both H3K36me2 and H3K36me3, with a higher affinity for H3K36me2 *in vitro* [31,108,109] whereas DNMT3B's PWWP preferentially binds H3K36me3 [31,107,110]. Although the basis of this difference in affinity is unknown, it is reflected in the localisation of DNMT3A and DNMT3B to H3K36me2 and H3K36me3 respectively in cells [30,31]. Cellular knockout of H3K36-methyltransferases also leads to redistribution of DNMT3A or DNMT3B [30,31].

The PWWP domain of DNMT3A is a common site of mutation in TBRS and AML (Figure 2). Systematic profiling of DNMT3A disease mutations revealed that 16/27 pathogenic PWWP mutants are unstable in cells [84]. Knock-in of the TBRS mutations W293Δ or I306N also results in reduced DNMT3A protein levels in mouse ESCs [22]. These findings emphasise the need to consider the effect of mutations on protein stability to understand disease mechanisms. This also suggests that even modest changes DNMT3A levels are sufficient to cause disease, a conclusion supported by neuronal phenotypes observed in heterozygous DNMT3A knockout mice [111].

However, some disease-associated DNMT3A PWWP mutations are stable, including R301W, observed in TBRS, and E342K, observed in breast cancer [84,101]. PWWP domains can also bind DNA [112,113] and the PWWP domain of LEDGF synergistically recognises H3K36me3 and both nucleosomal DNA gyres [114]. Indeed, the binding of DNA by PWWP domains was first recognised through analysis of DNMT3B [115] and DNMT3A was subsequently shown to bind DNA [108,116]. At present the PWWP domains of DNMT3A and DNMT3B have been absent from the nucleosome-adjacent density in cryo-EM studies [27,35] and as such it is unclear whether they engage DNA in the same manner as LEDGF. However, their DNA binding property is affected by disease-causing mutations, R301W was reported to decrease DNA binding of DNMT3A's PWWP *in vitro* whereas E342K increased it [101].

In contrast with the broadly distributed destabilising and catalytically inactive mutations seen in AML and TBRS, HESJAS mutations cluster around the aromatic cage of DNMT3A's PWWP [22] (Figure 3). Paraganglioma mutations are similarly distributed [25]. A recent case study reported a HESJAS patient with paragangliomas emphasising the link between the two conditions [117]. W330R abolishes H3K36me2/3 binding *in vitro* [22,108] and affects localisation to H3K36me2 in cells [118]. Some HESJAS and paraganglioma mutations are also reported to affect DNA binding by DNMT3A's PWWP [25]. The HESJAS mutation W330R increases DNA binding *in vitro* [101] whereas the paraganglioma mutation K299I decreases DNA and H3K36me2/3 binding *in vitro* [108]. The observations of both gain and loss of DNA binding caused by DNMT3A mutations in different disorders means that the significance of PWWP-mediated DNA binding in the mechanisms underpinning diseases remains unclear.

The PWWP domain of DNMT3B has been implicated in the pathogenesis of ICF1. In mouse ESCs, gene body methylation is dependent on DNMT3B recruitment to H3K36me3 [119,120]. Altered gene body methylation is also reported in ICF1 patient-derived cells [121] supporting the hypothesis that mutations affecting DNMT3B's PWWP-mediated recruitment to H3K36me3 could cause ICF1. Indeed, the sole ICF1 PWWP mutation, S270P [122], was reported to severely decrease interaction with H3K36me3 [30,107]. However, S270P has recently been shown to drastically reduce protein stability both *in vitro* and in cells [34] suggesting that the primary consequence of the mutation is loss of function, like other ICF1 mutations, rather than decreasing the interaction with H3K36me3.

DNMT3A's and DNMT3B's PWWP domains were proposed to mediate heterochromatin localisation [123,124]. However, this is likely an indirect effect; stable DNMT3B PWWP aromatic cage mutations (W263A and D266A) or those that decrease PWWP DNA binding caused increased localisation of DNMT3B to H3K9me3-marked constitutive heterochromatin and increased DNA methylation [34]. This parallels the hypermethylation of Polycomb-marked facultative heterochromatin observed in HESJAS and paraganglioma and suggests that loss of proper PWWP function causes redistribution of DNMT3A and DNMT3B from H3K36-methylated regions to other parts of the genome.

### N-terminal region

The N-terminal regions of both DNMT3A and DNMT3B are predicted to be largely disordered and is the region with the lowest sequence similarity between the proteins [41]. These regions have also undergone diversifying selection throughout evolution [125]. Hence, it was hypothesised that the N-terminal regions might mediate recruitment of DNMT3A and DNMT3B to different genomic regions [68]. However, for many years these regions remained little characterised. Early reports suggested that DNMT3A's and DNMT3B's N-terminal regions could bind DNA and anchor them to chromatin [30,126,127]. Recent studies have begun to dissect the function this region plays recruitment to both facultative and constitutive heterochromatin, defining new recruitment pathways that are important in disease.

The first study implicating the N-terminal region of DNMT3A, which is only present in the longer isoform DNMT3A1 (Figure 2), to specific genomic regions demonstrated that it was responsible for localising DNMT3A1 to facultative heterochromatin regions marked by the Polycomb-associated modification H3K27me3 in mouse ESCs and differentiated neurons [41]. Further dissection of DNMT3A1's recruitment to Polycomb-marked regions, demonstrated that this is mediated by interaction with H2AK119ub deposited by Polycomb-repressive complex 1 [128] rather than H3K27me3 [40,118]. A ubiquitin dependent recruitment (UDR) region was identified in DNMT3A1's N-terminal region and is required for localisation of DNMT3A1 to H2AK119ub [40,118]. Removal of H2AK119ub ablates DNMT3A1 recruitment to these regions [118], as does mutation of the UDR [40].

Three recent studies have reported cryo-EM structures demonstrating how DNMT3A1 specifically engages with H2AK119ub-marked nucleosomes [27–29]. These structures also revealed that the N-terminal region of DNMT3A1 interacts with the acidic patch on the surface of the nucleosomes, particularly Arg-181 [27–29]. The mutations R181C and A192E reduce binding to nucleosomes generally *in vitro* [27]. While these specific mutations have not been observed, mutations of Arg-181 and Ala-192 are reported in TBRS [129] and cancer [130,131]. In cells, disruption of these residues does not affect protein stability but reduces DNMT3A1's ability to repress transcription using a reporter assay [84] and its localisation and activity on chromatin [28,29].

Missense mutations in the UDR region of DNMT3A1's N-terminal region are also absent from the normal population [27] suggesting that interaction with H2AK119ub and the acidic patch is developmentally important. In mice, deletion of the N-terminal region of DNMT3A1, containing the UDR, retains normal DNMT3A expression but causes viability and behavioural defects suggesting the UDR is required for normal neural development and function [40].

**Figure 4.**
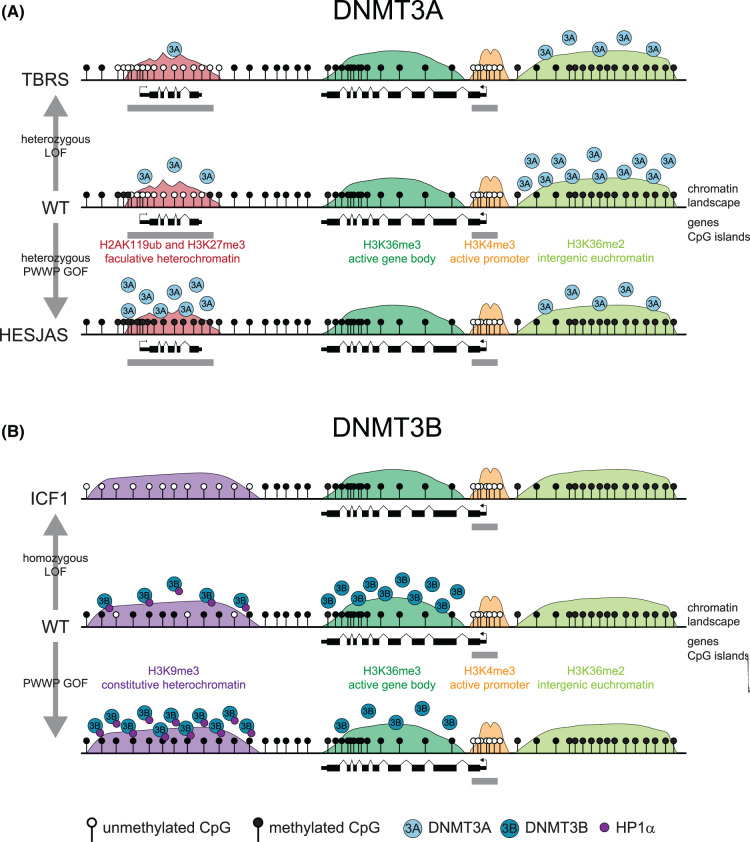
Disease mutations unbalance DNMT3A and DNMT3B recruitment. Schematics showing how the genomic distribution of DNMT3A and DNMT3B alters in human genetic disease relative to the chromatin landscape. (**A**) DNMT3A normally predominately localises to H3K36me2 which is associated with intergenic euchromatin [31]. TBRS heterozygous loss of function mutations results in focal hypomethylation at facultative heterochromatin marked by H2AK119ub [59,60]. Heterozygous gain of function mutations in HESJAS result in accumulation of DNMT3A and DNA methylation in these same regions [22,118]. (**B**) DNMT3B localises to H3K36me3 at transcribed gene bodies [30]. Homozygous loss of function mutations cause ICF1 and hypomethylation of constitutive heterochromatin marked by H3K9me3 [63]. Gain of function mutations result in accumulation of DNMT3B and DNA methylation in these same regions [34]. Abbreviations used TBRS, Tatton-Brown-Rahman syndrome; HESJAS, Heyn-Sproul-Jackson syndrome; ICF1, immunodeficiency-centromeric instability-facial anomalies type 1; LOF, loss of function; GOF, gain of function; PWWP, Pro-Trp-Trp-Pro domain.

The N-terminal region of DNMT3A1 plays a role in HESJAS and paraganglioma where PWWP mutations disrupt interaction with H3K36me2, redistributing DNMT3A1 to H2AK119ub-marked regions and causing DNA methylation gains [22,61,118]. This hypermethylation is reduced by mutations in the UDR motif affecting interaction with the acidic patch or H2AK119ub [28]. Taken together, this leads to a model whereby, in HESJAS and paraganglioma, DNMT3A1 recruitment is unbalanced (Figure 4A) [118] causing hypermethylation [22,28] through a UDR-dependent interaction with H2AK119ub and the nucleosome acidic patch. This epigenetic switch occurs at developmental genes and is accompanied by alterations in gene expression dynamics during neuronal and adipocyte differentiation in W330R mutant cells [22,28]. The altered gene expression dynamics could be explained by the hypothesis that Polycomb-mediated repression is labile [132,133] whereas DNA methylation represses gene promoters stably [134].

The N-terminal region of DNMT3B was recently shown to play a similar role in chromatin recruitment, except in this instance to H3K9me3-marked constitutive heterochromatin, which is hypomethylated in ICF1 [135]. DNMT3B interacts with heterochromatin protein 1α (HP1α) [136,137], which binds H3K9me3 and interacts with multiple proteins to heterochromatin [138]. This interaction requires the N-terminal region [34]. In parallel to the effect of HESJAS mutations on DNMT3A, PWWP domain mutations redistribute DNMT3B to H3K9me3-marked regions (Figure 4B) and DNMT3B activity in these regions is dependent on the N-terminal region [34]. At present no mutations to the N-terminal region have been reported in ICF1, but this work suggests the N-terminal region is important for methylation of a key compartment disrupted in ICF1.

Taken together, these recent studies suggest there is a delicate balance of different recruitment activities of both DNMT3A and DNMT3B, disrupted in a number of different diseases states.

### DNMT3 oligomerisation

**Figure 5.**
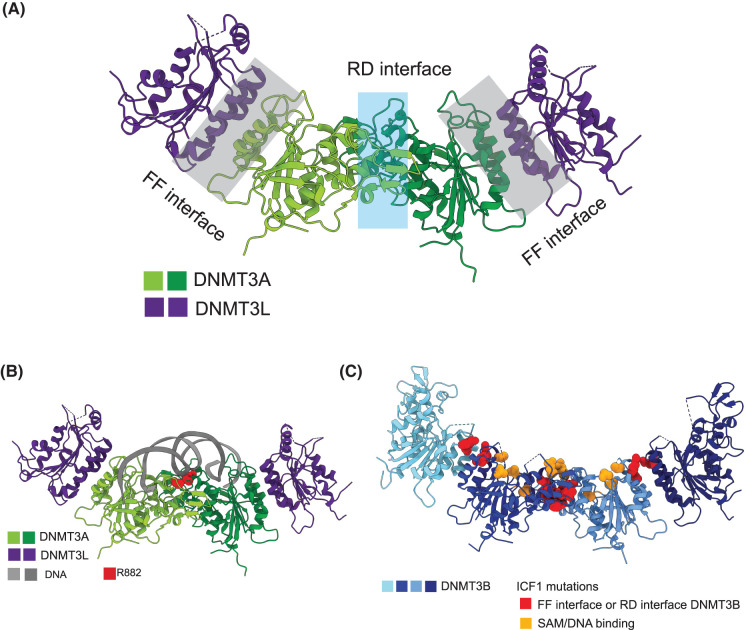
Oligomeric structure of DNMT3 enzyme methyltransferase domain. (**A**) Overview of structure of DNMT3A:DNMT3L methyltransferase domain tetramer indicating position of FF and RD interfaces. Structure from Protein Data Bank (PDB) accession = 6W8B [87]. DNMT3A catalytic domains indicated in green and those of DNMT3L in purple. Central RD interface highlighted in blue, peripheral FF interfaces between DNMT3A:DNMT3L highlighted in grey. (**B**) Structure of DNMT3A:DNMT3L methyltransferase domain tetramer in complex with DNA, showing position of Arg-882 mutation hotspot at the RD interface and contacting DNA backbone. Arg-882 is highlighted in red. (**C**) Structure of DNMT3B methyltransferase domain tetramer showing different types of ICF1 mutations. Structure PDB accession = 7V0E [88]. Different DNMT3B protomers of oligomer in different shades of blue. Mutations affecting SAM (cofactor, methyl-donor) and DNA (substrate) binding highlighted in orange. Mutations affecting FF or RD interface highlighted in red. All figures of structures generated using ChimeraX [139].

The methyltransferase domain of DNMT3A and DNMT3B mediates oligomerisation of the proteins through two interfaces, the hydrophobic (FF) interface and the polar (RD) interface (Figure 5A). The FF interface is named due to the stacking interaction of two phenylalanine residues which are key to this interaction interface [69]. In contrast, the central RD interface is stabilised by a Hydrogen-bond network, between an arginine and aspartate [69]. The RD interface is also involved in catalysis, linking oligomerisation to activity [69]. DNMT3A and DNMT3B methyltransferase oligomers have been primarily characterised as tetramers [69]. The adaptor protein DNMT3L lacks the RD interface and is found on the outside of hetero-tetramers with two central DNMT3A or DNMT3B molecules [69,76,140]. This has also been shown for the catalytically inactive DNMT3B3 [35]. DNMT3A and DNMT3B are also hypothesised to form higher order oligomers via the RD and FF interfaces, as previously reviewed [141,142]. In support of this, a recent cryo-EM structure of DNMT3B reported trimeric and hexameric states in addition to a tetramer [103] and a DNMT3B homo-tetramer assembles into supramolecular helical assembly in a crystal structure [88].

The oligomerisation of DNMT3A and DNMT3B raises the question of how numerous domains with unique chromatin-reading specificities are integrated, something that becomes even more complex in the context of hetero-oligomeric complexes. DNMT3B3 or catalytically dead DNMT3B, can recruit DNMT3A to H3K36me3 marked regions [51,143] providing some insight into the behaviour of hetero-tetrameric complexes in the context of whole proteins. Mice with catalytically inactive DNMT3B mutations are viable and present few methylation changes compared with DNMT3B knockouts [144,145]. This suggests the chromatin recruitment role of DNMT3B as an accessory factor for active DNMT3A is required for normal development. In a recent study, mutation of both the DNMT3A-ADD and DNMT3L-ADD had greater decrease in global CpG methylation during gametogenesis than the individual mutations [99]. This suggests both protein's ADD domains contribute to the function of DNMT3A:DNMT3L complexes. Given that splicing of DNMT3B3's methyltransferase domain generates a novel interface that interacts with the acidic patch of the nucleosomes (Figure 2) [35], the recent finding that DNMT3A1's UDR also interacts with the acidic patch [27–29], means that it is unclear how competing acidic patch binding is balanced in the context of DNMT3A1:DNMT3B3 complexes.

Mutations affecting DNMT3A oligomerisation are also found in disease. As well as contacting DNA, Arg-882 is located close to the RD interface (Figure 5B). DNMT3A is reported to have a cooperative polymerisation mechanism that differs from DNMT3B [71]. Recent structural characterisation of R882H AML mutation in the context of the DNMT3A homo-tetramer suggests that the mutation increases interface stability, intermolecular contacts and results in polymerisation beyond the level of tetramers (Figure 5) [146,147]. Using R676K or M674T + R676K mutations that mimic DNMT3B, Lu et al. [146], also demonstrated that R882H oligomerisation defects could be rescued while preserving its altered flanking sequence preference. These mutations also rescued cytokine-independent growth in erythroleukemia cells, arguing that the oncogenic effect of R882H is mediated by its effect on oligomerisation [146]. Another frequent DNMT3A cancer mutation R736H is located at the FF interface and is reported to make this interface more flexible and stimulate DNMT3A activity in wildtype:mutant hetero-complexes [148].

Similarly, ICF1 mutations are found in the DNMT3B oligomerisation interfaces. ICF1 mutations at the RD interface H814R, D817G, V818M disrupt dimerisation and DNA binding resulting in reduced catalytic activity [91]. Analysis of the homo-oligomeric structure of the DNMT3B methyltransferase revealed differences in the interplay between the FF interface and catalytic loop in DNMT3B homo-tetramers compared with DNMT3B:DNMT3L hetero-tetramers [88]. Several ICF1 mutations are located at the FF interface (Figure 5C) and the interface of the DNMT3B homo-oligomer was also more sensitive to the mutations L664P, L664T and R670Q than that of DNMT3B:DNMT3L hetero-tetramers [88]. Given that DNMT3L expression is largely restricted to early development and germ cells [53,56,149], this observation raises the possibility that ICF1 mutations may affect DNMT3B activity differentially as development proceeds.

These observations suggest that oligomerisation is important for DNMT3 function and that disease mutations must be considered in the context of oligomers.

## Conclusion

Characterisation of disease-causing missense mutations in *DNMT3A* and *DNMT3B* have greatly advanced our understanding of the regulation and genomic recruitment of these enzymes and motivated molecular studies to understand their function. This reveals that mutations impact a diverse range of DNMT3A and DNMT3B molecular functions causing different DNA methylation changes and diseases. In particular, recent efforts have shed light on the functions of the previously poorly characterised and disordered N-terminal regions of both proteins defining new heterochromatic recruitment pathways. They also suggest that different DNMT3A and DNMT3B recruitment activities are finely balanced in cells and this balance is disrupted by disease mutations. At present, however, it is unclear how this balance is regulated normally. Recent work also emphasises that DNMT3A and DNMT3B oligomerisation is altered by disease-causing mutations. However, currently this oligomerisation has largely been studied *in vitro* and we do not understand the range of oligomers present *in vivo* nor how the multiple recruitment activities within these oligomers are balanced and regulated. Another emerging theme is that many disease-causing *DNMT3A* and *DNMT3B* missense mutations are destabilising. This highlights the need to characterise the effects of disease-associated mutations on protein stability, as failure to uncover a destabilising effect can lead to incorrect conclusions being drawn about the primary mechanism through which a mutation causes disease. Overall, the molecular understanding generated by this collective effort provides a strong platform from which to determine the mechanisms responsible for disease. It will likely prove important in determining how DNA methylation changes occur in cancer, where the role of DNMT3A and DNMT3B remain unclear despite reported mis-regulation, altered splicing and mutations. Ultimately, the mechanistic insights derived from the study of patient mutations could also lead to therapies that improve patient outcomes.

## Perspectives

- Disease mutations in *DNMT3A* and *DNMT3B* have motivated studies to uncover the mechanisms governing the recruitment, regulation and function of these proteins. This provides a paradigm to understand the role that DNA methylation and chromatin alterations play in cancer and other human diseases.
- The different domains of DNMT3A and DNMT3B regulate their activity and recruitment by recognising specific chromatin features and are diversely disrupted by disease mutations. Many disease-causing mutations have been shown to destabilise the proteins, highlighting the importance of assaying this possibility for disease-associated mutations to avoid drawing incorrect conclusions as to the mechanism through which they might cause disease.
- Recent findings have emphasised the importance of the balance between different DNMT3A and DNMT3B recruitment mechanisms, particularly the N-terminal region and PWWP, but the regulation of this balance is poorly understood. How different recruitment and regulatory activities are co-ordinated in the context of DNMT3 oligomers is also poorly understood.

## Open Access

Open access for this article was enabled by the participation of University of Edinburgh in an all-inclusive *Read & Publish* agreement with Portland Press and the Biochemical Society under a transformative agreement with JISC.

## Abbreviations

AML: acute myeloid leukeamia
ESC: embryonic stem cell
SAM: *S*-adenosyl methionine
TBRS: Tatton-Brown-Rahman syndrome
UDR: ubiquitin dependent recruitment
